# American Academy of Dental Science

**Published:** 1897-10

**Authors:** 


### AMERICAN ACADEMY OF DENTAL SCIENCE.

THE regular monthly meeting of the American Academy of Dental Science was held at Young's Hotel, Boston, Wednesday evening, February 3, President Andrews in the chair. Subjects for discussion:

I. Retaining Appliances in Orthodontia.

II. Dental Materia Medica.

### DISCUSSION.

*Dr. Briggs.*—Mr. President and gentlemen, that which occurs to me more and more in practice—and it is quite opposite to views that I once held—is that the dentist must more and more fit himself to practise medicine, in the line of prescribing, to a certain extent, drugs for his patients. In the old days, before the hypodermic syringe, and when certain drugs were the only ones to be found in the dentist's armamentarium,—when morphine was the only analgesic, and that was too dangerous to use unless one could watch its operations,—it did not seem proper for a dentist to prescribe the use of drugs where the circumstances would be such that the dentist could not see the actual result, and thus protect the patient from any harm which might result. Times have changed; there has been an influx of the coal-tar products, and of combinations of cocaine, which, whether they are dangerous or not, the laity have no hesitation in using, sometimes in quantities and under conditions where a dental practitioner would not.

The thing that is forcing us into it, perhaps more than anything else, is the fact that the physician refuses to be educated in dental matters. Time goes on, and no attention is paid in the medical schools to teaching anything about the mouth or the teeth, while the dentist himself is getting broader, and is reaching out more into the domain of medicine. This tendency of the physician to ignore the dentist and his work is exemplified to us every day, and he probably knows as little to-day about the effects of these coal-tar products on the pains and inflammation with which we come in contact as the majority of the patients themselves.

Apropos of the feeling of the physician that the domain of the dentist is not in his line, I will mention a case of a physician who was called to a lady who was confined to her bed with an abscessed tooth, the abscess having formed in the roof of her mouth. The physician observed it, and said, "You must send for your dentist right away." When asked if he could not attend to it and relieve her suffering, he said no, it was something for the dentist to do. A messenger was sent to me, who explained the case, and I sent an associate, who lanced the abscess, and gave directions for the treatment until the lady could visit the dentist. The ridiculousness of a physician stating that the treatment of that abscess was different from an abscess anywhere else shows their limitations in matters connected with the mouth. Of course, we know that the principles of treating an abscessed tooth, as far as the immediate emergency treatment is concerned, are just the same as those which underlie the treatment of an abscess in any other part of the body. And so it is when a person is suffering severely from pulpitis, pericementitis, or alveolar abscess, and the physician is sent for; he is perfectly helpless, and does not know how to go to work to relieve the patient, and unless we step in and give the proper treatment the patient must suffer in consequence.

The hypodermic syringe has brought obligations upon us. If we give anæsthethics, we must be able to use the hypodermic syringe to revive patients from shock or from being overcome by chloroform or ether; then we very easily use it in many other conditions to relieve pain and assist operative interference. Our knowledge of the use of the hypodermic syringe in many of the minor surgical operations about the mouth has brought about an enlargement of our field, and has led us to do what we have hitherto not attempted, because it involved the general anæsthesia of the patient.

So, to sum up, it seems to me that instead of teaching, as I have always done, that we wanted the dentist to know materia medica in a broad and general way, as a matter of education, and to point out at times the necessities of the patient to the family physician, it has come to the point that, if the dentist is to take full charge of his department, he must be capable and fitted to apply the entire materia medica,—not "dental materia medica;" there is no dental materia medica; materia medica is the same for the dentist as it is for the physician, and the same principles that underlie the treatment in any part of the body apply to all the conditions with which the dentist meets. I hope what I have said, Mr. President, will start a discussion.

*Dr. Eames.*—I have brought for your inspection a few of the advertising leaves from the January number of *Items of Interest.* On one of these "Dr. Hayes's Improved Process of Anæsthesia" is advertised, and I know that very many dentists are deceived, and influenced to pay one hundred dollars for an "office right" use of preparations, of the composition of which they are utterly ignorant, and to administer them to patients for the production of anæsthesia. It is a serious thing to administer any anæsthetic: the patient is rendered unconscious; the powers of life are, one by one, suspended, approaching the very doors of death, yet this secret mixture is held up to the profession as entirely harmless.

I think that we ought to put the stamp of condemnation on these and other secret remedies, such as "Uzane," "Paralesia," "Noxall," and others, and have it understood that this society disapproves of advertising them in professional journals. Although our society has a record of action against secret remedies, it seems necessary to act again, and I hope something will be done to-night.

I would now like to refer to an old remedy that has been revived by myself,—that is, rapid breathing as a pain obtunder. We all know that Dr. Bonwill was the first to introduce it. I happened to be present when he made a demonstration in the Pennsylvania Hospital in 1876, but I have never used it until within the last three years. One of my first operations was the removal of a sebaceous cyst. I made a free incision across it, turned out the sac, and dressed it, without the slightest pain to the patient. I have extracted teeth in sufficient numbers to perfectly demonstrate its efficacy, and have had no ill results. I do not use this method indiscriminately, but select the patient.

Now, a word in regard to the coal-tar series of remedies. We receive numerous samples and circulars regarding these preparations, but we do not always know their composition. I think it is much better, in such cases, to prepare our own remedies. For instance, ammonol is a good sedative theoretically, but practically it is unreliable. I would prefer to prescribe acetanilide with the proper amount of ammonium carbonate, freshly prepared.

We have a new product, eucaine, which is advertised as a substitute for cocaine. I believe we should test this more fully and for a longer time before accepting it as perfectly harmless. I have some reasons to doubt that it is perfectly harmless, at the same time I have used it in quite a number of instances during the past eight months. In one case I used it freely to extract five roots of teeth at one sitting, injecting it very freely, without any after-effects. I do not think that, applied to mucous membranes, it is quite as effective as cocaine.

I have been making some experiments upon animals, which I have not yet completed. As far as I have gone, however, eucaine seems to be more toxic than cocaine, killing a frog which had recovered from a similar dose of cocaine.

*Dr. Banfield.*—Will Dr. Eames tell us what per cent. and how much he injects of the eucaine?

*Dr. Eames.*—Usually a four-per-cent. solution.

*Dr. Piper.*—I would like to ask Dr. Eames if he has had an opportunity to notice the after-effects,—the swelling which he said occurred the next day?

*Dr. Eames.*—You will remember I mentioned in passing that it is claimed that there is an infiltration of tissue, and it is admitted that a swelling may occur the next day, which is painless and harmless. In my own cases I have not had marked swellings.

I do not feel that I can agree with a recent statement in one of our journals in regard to the Schleich method of infiltration, which is an excellent method of producing local anæsthesia. The statement was made that with a solution of one grain of cocaine to the ounce you might inject an ounce without any fear of the result. That would mean a grain of cocaine, and I do not think that a grain of cocaine should be injected into the system with impunity by the Schleich or any other method.

*Dr. Adams.*—I would like to ask Dr. Eames if he has ever tried the rapid breathing in the excavating of teeth.

*Dr. Eames.*—Not enough to speak definitely about it.

*Dr. Wilson.*—I would be pleased if Dr. Eames would speak a little more in detail in regard to the method of rapid breathing. I have heard it spoken of a great many times and never paid much attention to it, but if there is really anything in it, I should like to know just what can be done. To be entirely candid, I never supposed it amounted to anything.

*Dr. Eames.*—I had in mind to bring a patient here and do a little operation, but I did not think that this would perhaps come into much prominence, and hesitated about taking the time of the

Academy. I feel that in regard to sensitive dentine it might not be used so much, because it is hard work to get many patients to co-operate. You have to use a good deal of energy yourself to get the patient to make sufficient effort to bring about the result; you have to constantly urge them to breath rapidly. Its very simplicity makes the patient unwilling to attempt it. You have to be convinced of its efficacy in order to influence them. Until I was convinced, I did not urge it, but now, unless the patient very decidedly objects, I insist upon it. It lasts a very short time, and if the operation is to be extended, you have to ask the patient to continue breathing.

*Dr. Payne.*—Dr. Eames spoke of selecting his patients. I would like to ask him if there are any dangers connected with it that it would be well to be prepared for?

*Dr. Eames.*—In case of a patient with weak lungs there might be a liability to strain, which might be productive of some injury.

*Dr. Stevens.*—I wish to substantiate all Dr. Eames has said in regard to rapid breathing. I know that it is wholly true, and the way it came to my attention first was perhaps twenty years ago; one time when the gas was all out of the tank and a patient came in to have a tooth extracted and wanted to take gas. After the first two or three inhalations the gas gave out. I knew it would be useless to attempt to explain matters, so I allowed the patient to continue breathing air instead of gas, and it had the same effect. I was talking to Dr. Bonwill one day, and told him about this, and he corroborated my statement very heartily. He told me how he had made the discovery himself in a very similar way, and while out walking one day he began to think over the matter, and got so interested that he sat down on the curb-stone and made a study of it right there in the street. He got very enthusiastic over it, as many of you may remember. The only trouble about it is to make the patients believe it. It is such a simple thing that they will not believe when you tell them that it is only necessary for them to breathe rapidly for a few minutes and you will be able to extract their tooth for them without pain.

*Dr. Clapp.*—I have been very indignant for a long time at the character of the advertisements which appear in the dental journals. I am glad that Dr. Eames has brought this matter up, and, just to test your opinion with regard to it, I will introduce resolutions, which you can think over and discuss, and if you wish, I will present them in the form of a motion.

"*Resolved*, That the officers of this Academy be instructed to draw up a resolution condemning the publication in all professional journals of unprofessional advertisements.

"*Resolved*, That this resolution be forwarded to the leading dental journals as expressing the sentiment of this Academy."

*Dr. Eames.*—I should like very much to see a motion of this sort carried. In talking with the head of the Consolidated Company here, he asked me how I liked the journal. I replied that its articles, contributions, etc., were very satisfactory, but to have them associated with the cuts and advertisements which appear in the journal I considered incongruous and unprofessional. He replied that the journal could not live without its advertising pages; and I have thought since that if we cannot support a dental journal without its being associated with these quack advertisements, we had better do without it altogether.

*Dr. Banfield.*—Who is to be the judge of the advertisements, as to whether they should be allowed to appear in a dental journal? What we might consider questionable advertisements they might say were all right.

*Dr. Clapp.*—It certainly might be difficult to draw the line, but it seems to me that an expression of this kind would be pointed enough for the journals to understand, somewhat, our sentiments. Now, Dr. Eames has made a remark which has impressed me very much,—that is to say, he has quoted a remark made by one of the officers of the Consolidated Company, that the "journals could not live without the advertisements." It is a very great question in my mind whether it is necessary to have so many journals live; the sooner some of them die the better. We do not need many journals to take care of all the choice dental literature that is presented for publication, and I do not see why we should feel any obligation or desire to support two or three in Boston, five or six in New York, seven or eight in Philadelphia, fifteen or sixteen in Chicago and the West. For my part, I do not see why they should be supported.

*Dr. Smith.*—This matter can be looked at from two stand-points: we look at it from a professional point of view, while the Consolidated Company view the matter from a business stand-point. They are serving, as they think, their customers. It is strictly a business transaction, and to the person in business all advertising is legitimate which can be safely carried through the United States mails.

The remedy for this evil is in our own hands. The support of these journals rests largely with the profession, because it is through the profession that the advertisers and publishers expect return for their outlay, and if there is anything we dislike about the journals, all we have to do is simply to refuse to take them, and refuse to publish anything in them, and the matter would soon adjust itself. If the profession would take concerted action in such matters as these, they could correct many things which they dislike. How difficult this is to accomplish, even among those we term the best and highest of the members of the profession! I have no doubt that the journal in question is a good one, but it is published in the interest of a dental manufacturing company, whose object is to bring the goods they manufacture before the dental profession. Now, we have a dental journal that is published by the profession and for the profession. I shall not attempt to say what success we are having, but I will say that so far the road to success has been up-hill, and why? Because many of the men who take the INTERNATIONAL DENTAL JOURNAL, and who will tell you that they believe in its objects, when they have a paper which they consider has some merit to it, go and publish it in the *Dental Cosmos*, because they want it to reach the greatest number of readers, and an article published in the *Dental Cosmos* is read by almost the entire profession. If these men would publish their papers and ideas in a strictly professional journal, the dentists, who are ready to support anything, would soon find out that it was for their interest to take these professional journals published by professional men, and the withdrawal of this literature from the trade journals would deprive them of their value, so that the profession would cease to support them.

*Dr. Clapp.*—I believe that, and I believe we should do more. I believe we should let these journals know our sentiments. And for that reason I have thought the method proposed would be an effective way of bringing it to their attention. It has been suggested that the advertising in the dental journals was a business matter in the same sense as that of the various trade journals. I do not think that is so. The advertisements in the various secular papers of ordinary business do not degrade anybody, but the advertisement in professional journals of unprofessional remedies and methods does degrade the dental profession, and it is one of the most pernicious examples that is presented to our young men as they enter into the practice of dentistry. I think our disapprobation of such methods should be made known as the sentiment of the American dentist, not the Boston, or the Massachusetts, or the New England, but the American dentist.

Mr. President, I move that the resolutions which I have read be adopted by the Academy.

*Dr Smith.*—I heartily second the motion. Certainly there can be no harm, but great good, in expressing a sentiment of this kind.

*President Andrews.*—I put this motion with a great deal of pleasure. It is rumored that the privilege of the publication of the transactions of one of our largest and most active dental societies has been sold to a trade journal for several hundred dollars, —I do not remember the exact amount. It seems incredible that a society of professional men should withdraw their support from a professional journal and transfer their proceedings to a trade journal for a money consideration, and it is with much pleasure that I put this motion, to adopt the resolution which Dr. Clapp has offered.

*Dr. Wilson.*—I believe the motion is a good one, and while it may appear to some that the matter of advertisements is something which should not concern us, yet it is in line with keeping up the standard of the profession. What our President has said about a dental society selling their transactions was news to me.

It seems to me that if we are earnest about this motion that has been made, we might consult with other societies and see what they will do. We ought to push the thing a little further than simply acting upon it ourselves. I heartily believe in it.

*President Andrews.*—The matter to which I referred has made such a stir that many of its most influential members have left the society. Are you ready for this motion?

Unanimous vote.

*President Andrews.*—We have two subjects for discussion this evening, on the first of which, "Retaining Appliances in Orthodontia," Dr. Smith will open the debate, but before speaking on that subject he has another matter which he would like to call to the attention of the Academy.

*Dr. Smith.*—Mr. President and gentlemen of the Academy, preparatory to speaking on stay-plates used in orthodontia I would like to present a case showing the value of the shadowgraph in our work. The case is represented in these two models. This one, marked B, shows the condition of the mouth at the time of the eruption of the upper right lateral incisor. The left central and left lateral came through in their proper places, and when the patient was about eight years of age the case came into my hands. All I could then know was that a tooth was presenting itself, there being a fulness of gum-tissue, and I understood that the lateral incisor had previously loosened, and that the patient's mother, or some one at home, had taken it out. The temporary central incisor was also very loose, and was extracted. The permanent lateral then erupted, and took a position inside the line pointing towards the central. Appliances were made to bring the lateral tooth outside of the lower incisors, and we waited for developments. This model shows the case in 1891, and we have watched with interest this case ever since, hoping all the time that the central incisor would soon come down. The other day the patient came in, being quite elated at seeing the point of a tooth directly over the lateral, thinking it was the central. A few days later the tooth had come through sufficiently for me to see that it was the permanent cuspid. The temporary cuspid was extracted, that being somewhat loose, and I was then very much in doubt as to what to do, having waited so long for the central to come down. I have not had time to have a cast taken showing the mouth just as it is at this moment, but this mark shows very well the cuspid tooth now picking through directly over the lateral. I got the consent of the patient to go with me to Dr. Clapp's and have a shadowgraph taken, and this work of Dr. Clapp's shows up as rather the best he has done, and we all know that he has accomplished some very good work in this line. From it you will see that there is a tooth here, the necessary right central incisor between the right lateral and the left central, that is turned half round. With this explanation, which it seems to me all that is necessary, I will pass these models about with this shadowgraph.

Now, as regards retaining appliances.

The remark that is frequently made that the correction of an irregularity is easier than the permanent maintenance of the results, I do not think is true. The disappointment which has resulted in many well-planned cases of orthodontia has been due to the too early application of retaining appliances. I think that is an error we all make. I made it myself, and by experience I have learned that after accomplishing a change in the teeth, if you allowed the appliance that did the work to remain for some time after the teeth have been brought to a satisfactory position, so that the new bone may be deposited, then the newly made appliances for retaining more readily holds the teeth in their changed position.

There is no question but what the best retaining appliance is a fixed appliance. To illustrate this, you take, for instance, a case of protrusion of the anterior teeth which have been brought into line.

If an appliance can be made by putting bands on the molars and cementing them to place, with a wire running from the molars around the front of the teeth, the ends being soldered to these bands, you have the ideal retaining fixture; but it is rarely that you can use these fixed appliances,—not because it is a mechanical impossibility by any means, but because patients object to retaining appliances that cannot be removed for certain important society events. Many of the patients for whom we are called upon to do regulating are young girls just going into society, and whenever anything of the kind has to be made, the families of the patients insist that it shall be something that can be removed when it is thought desirable to do so, and so I find that to accommodate this great demand of society I am obliged to devise some appliance which the young lady can remove just before stepping into the ball-room, and put on again immediately after returning home. Of course it takes longer for the teeth to become fixed in this way, because the tendency of the teeth to go back to their old position will assert itself upon the removal of the pressure, even if only for a few hours, until such time as the new tissue can hold them firmly in the desired position, and yet that is all we can do under the circumstances. But, as I said before, the fixed appliance is without question the ideal appliance, and the removable retaining appliances are, as a rule, better made with some form of vulcanite with metallic attachments. If a crib appliance is used, which many seem to prefer, they have to be made quite heavy, for if they are not, you will find that patients in taking them out and putting them in are apt to get them out of place, and when they do not succeed in getting them into place without much trouble, they will be laid aside.

Of course, it should be the aim in making a retaining fixture to have the smallest possible contact with the teeth in order to prevent the possibility of decay. The retaining appliance shown here by Dr. Baker a few years ago was an ideal one of its kind. It requires great skill to make such an appliance and have the bearings accurate, and in cases where teeth have only been moved out or in, an arrangement of that kind is admirable. The idea is a modification of one that is presented by Dr. Farrar in his work on Irregularities, and for the very skilful adaptation of the idea to his individual case Dr. Baker certainly deserves a great deal of credit.

In regulating cases, where we have simply moved an instanding incisor out over the lower incisors, many times the occlusion itself is sufficient to retain the tooth in position, but sometimes you will find that after the correction of the irregularity your incisor still drops back a little to an occlusion with the lower. Now, such a case as that could be nicely retained by a simple fixture, say a band around the tooth with wire projections resting on the adjoining teeth, and yet you will find it is the desire of most patients to avoid wearing such an appliance, and your only recourse, therefore, is to again resort to an application of the vulcanite plate, with little wire projections behind the tooth keeping it from moving back.

The Watt plugs, which writers upon this subject allude to, is a method that I do not think is very much employed. I confess I never saw a case except in the pictures. When you look at the pictures and read the description of them, it would appear to be a practical thing, but to my mind it is not so feasible as it would seem. If you are intending to hold a tooth in place with a Watt plug, it is necessary that there should be a cavity. If there is not one there already, you must drill into the sound tooth and make one, and I very much doubt the expediency of mutilating a tooth for the sake of making a place to hold this plug, and I think, notwithstanding all that has been said to the contrary, that a vulcanite plate is far superior to the Watt plug method of holding teeth in place.

There is another point in regard to this matter of retaining appliances of which we often lose sight, and that is the tendency of regulated teeth to return to their old form of irregularity. There seems to be a natural tendency in that direction which in some cases it seems to be almost impossible to overcome. The teeth are moved into place; retaining appliances are put on and worn by the patient for a year or more, and then taken off, and back the teeth go to their old position, and the entire work is regarded as a failure, when really the only error was in the fact that the retaining appliance was removed too soon. In other words, there are many cases of corrected irregularities where the apparatus for retaining the teeth in their changed position should have been a retaining appliance for life,—a permanently fixed appliance, which should be allowed to remain in the patient's mouth. That is what we lose sight of. If I understand the object of our work in prosthetic dentistry, it is in part to make people better looking. If it is professional to extract natural teeth for the purpose of supplying artificial teeth and improving a person's appearance, it is certainly quite as professional to make a fixed retaining appliance by cutting into the teeth and anchoring them by wires to hold them in the desired position, with the same end in view. There are cases where we are forced to do this. Take, for instance, a case where we have a protrusion of the incisor teeth in a patient somewhat advanced in years, say about thirty, and you have drawn them back into line and wish to retain them. The usual method of retaining in such a case is to drill into the palatal surface of the teeth and set wire retainers which rest on the adjoining teeth, so that it is impossible for those teeth to move out. Now, the tendency of those teeth to move out, especially in a person of that age, is something astonishing. I recollect a case of the kind where two central incisors had been drawn back and wire retainers, resting on the laterals, were put in, supposing the laterals would be sufficient to hold them. They not only moved forward themselves, but they took the laterals along with them. A new appliance had to be made for correcting, and the same kind of a fixture was made for retaining, except that this time it was extended to include the cuspid tooth, and there was no further trouble.

The many applications and modifications of these crib arrangements are to be governed by the individual case and the ingenuity of the operator. With so many different appliances pictured in the books, I think one shows quite as much skill in the proper selection to suit the case in hand and in carefully adapting it as he would if he set about to originate something new himself. In many instances, after spending a great deal of time and thought on some particular operation, he will find that his ingenious production is simply an old idea worked over, with some modification of his own, adapting it to the individual case.

The disadvantage, of course, of all vulcanite work for retaining is that there is so much surface in contact with the teeth that you have a great liability to decay, at least so it is claimed, and yet, out of a great many such appliances that I have put on, there have been very few, comparatively, with which I have had any trouble on account of decay; in fact, at this moment I can recall but one, and that was a case where a dentist extracted the sixth-year molars because they were badly decayed; but as the case turned out, it would have been better to have kept the molars in and crowned them. The arch had been spread to correct an irregularity, but it would not remain, and it was necessary for the child to wear a vulcanite plate constantly, and in that case the teeth decayed; but it was the only one that I remember having seen in which decay resulted from a vulcanite plate for retaining.

I think I have briefly covered the general principles of retaining appliances, but after the discussion has started, it may bring something to my mind of which I will be glad to speak later.

*Dr. Ainsworth.*—I wish to say "amen" to the remarks of Dr. Smith. I agree with him in regard to leaving on the appliance with which the teeth have been moved for some time after they are in a satisfactory position before undertaking to make a permanent retaining fixture, but I find a common reluctance among patients to wearing it after the teeth are in position,—that is, the appliance with which they have been moved,—and I have been many times influenced by their wishes, much to my regret.

There are four things, to my mind, that are important in a retaining apparatus: it should be positive; it should be as cleanly as possible; it should be comfortable; and it should not present an unsightly appearance.

As Dr. Smith has said, we find among the class of people for whom we are called to practice this specialty a reluctance to wearing anything that is conspicuous, and we are constantly called upon to exercise our ingenuity in making something that shall be as little noticeable as possible; but such appliances have in the main proved unsatisfactory.

A fixed appliance, it seems to me, is far preferable to a removable one, because it is much more positive, and you are sure that it is being worn twenty-four hours a day, while a removable one may be in the pocket twelve hours a day, in which case it will take a long time for the teeth to become fixed, if, indeed, they do at all. They will be very likely to go back to their former position when the appliance is finally removed.

An appliance should be made so as to have as little contact as possible with the teeth, to prevent injury and facilitate cleanliness.

I hold in my hand a case for which I had made this gold retaining-plate, designed to be unconspicuous and to please the patient. The arch had been spread and the front teeth drawn in,—a case of "thumb-sucking." This plate was put in last spring, and I discarded it in the fall as practically a failure: it was not sufficiently positive; then, too, we needed all the room between the incisors to hold them down to place, and the little gold projections on the plate coming between the incisors kept them slightly out of line. This case was one in which the patient seemed unusually disturbed lest she should have to wear something which would show too much. I finally hit upon this compound, and solved the problem in the following way, which is proving eminently satisfactory; and one of the requisites was that she should be able at times to leave off the part which was noticeable:

I made gold bands for the two cuspid teeth, also for the two sixth-year molars; shaped a sixteen-gauge platinized gold wire to correspond with the arch and rest against the palatal surfaces of each tooth; this arch band was soldered to the bands encircling the cuspid teeth, and the ends engage in tubes soldered to the palatal surface of the bands encircling the molars; the molar bands were then cemented into position, after which the cuspid bands were cemented in place, carrying with them the arch band, the ends of which engage in the short tubes on the molar bands; this serves to hold the bicuspids and molars from dropping in, and to hold the incisors in. I soldered short pieces of gold tubes in a perpendicular position to the distal buccal surfaces of the bands encircling the cuspids, then, forming a smaller platinized gold wire to fit the labial surfaces of the front teeth, I bent the ends at right angles so as to engage in the little tubes attached to the cuspid bands; this front band can be taken off and snapped back in place at will. When off, nothing shows but the bands on the cuspids. Of course, in cases of this kind the front teeth must be held down in position a long time. In time I anticipate the patient will get along all right by wearing the band at night only.

*Dr. Cutter.*—I have nothing special to say upon the subject, which has already been so fully discussed.

I would, however, state that I have rarely seen any injurious effects from vulcanite and wire retaining-plates coming in contact with the teeth, yet I am always particular to instruct patients upon the importance of keeping such appliances as clean as possible.

*Dr. Smith.*—I will simply state that the case where Dr. Ainsworth spoke of having made a satisfactory appliance by placing anchor bands upon the cuspids, with a wire passing along the palatal surfaces of the anterior teeth, was good as far as it goes, but it only goes to the cuspid, and that is fatal. In the great majority of cases you would find that people would object to the bands on cuspids; they do not want to have any gold showing. And then, again, the objection might be offered that the wire on the inside touches the palatal surface of the teeth; if fairly large, I should fear the chances of decay in these much more than from a vulcanite plate that was removable. I have seen a number of cases where two wires were used,—one passing along the inside of the teeth, being ligated to the cuspid, and the other passing on the outside, ligating the tooth which was to be carried into line; and

I have seen more harm done in that way than could possibly have been done by a vulcanite plate pressing against the palatal surfaces of the teeth.

*Dr. Ainsworth.*—I would say, in answer to Dr. Smith, that the band is not a flat wire, as it appears in the cast here. It is a round wire, and does not touch the teeth except in spots that will be in the future entirely exposed to friction, where there is no ordinary tendency to decay and no fear of decay. The teeth are good and strong, and the position of the band is such that it is cleanly. If there were ligatures worn here, there might be trouble, but I have no expectation at all of this band doing any harm.

